# Effect of bioceramic sealers in enhancing root canal healing

**DOI:** 10.6026/973206300210866

**Published:** 2025-04-30

**Authors:** Shankar S., Rajul Vivek, Nitu Chaubey, Vinay Rao, Sagar Hiwale, Nandhita S.K

**Affiliations:** 1 Department of Conservative Dentistry & Endodontics, NSVK Sri Venkateshwara Dental College & Hospital, Bannerghatta, Bengaluru, Karnataka, India; 2 Department of Prosthodontics and Crown & Bridge, Purvanchal Institute of Dental Sciences, Gorakhpur, Uttar Pradesh, India; 3 Department of Conservative Dentistry and Endodontics, Purvanchal Institute of Dental Sciences, Gorakhpur, Uttar Pradesh, India; 4 Department of Conservative Dentistry, Endodontics and Aesthetic Dentistry, AMC Dental College, Ahmedabad, Gujarat, India; 5 Department of Conservative Dentistry & Endodontics, Inderprastha Dental College & Hospital Ghaziabad, Uttar Pradesh, India

**Keywords:** Bioceramic sealers, root canal healing, endodontics, periapical lesions, bioactivity, dental sealers, pain reduction

## Abstract

Root canal treatment requires optimal sealing to stop infection from recurring while aiding the healing processes. Therefore, it is of interest to examine the analysis of bioceramic and conventional dental sealants using 100 patient samples within a period of six months. Bioceramic sealers achieved better results since 90% of patients experienced periapical healing compared to 75% in the conventional group. The bioceramic group excelled in both reducing pain and attaining superior radiographic sealing quality. Thus, healing progresses better when dentists use bioceramic sealers which enhance root canal therapy according to expert recommendations.

## Background:

Root canal therapy is a vital procedure in modern dentistry aimed at saving infected or damaged teeth by eliminating bacterial infections within the root canal system and preventing reinfection. Success in root canal therapy largely depends on the quality of the root canal filling, which must effectively seal the canal space to prevent bacterial ingress. The materials used in this process, particularly root canal sealers, play a crucial role in determining the long-term outcome of the treatment [1, 2]. Bio-ceramics, with excellent bioactivity and biocompatibility, have been widely used in dentistry, particularly in endodontics [3]. In recent years, bioceramic sealers have emerged as a promising alternative due to their superior sealing ability, bioactive properties and biocompatibility. Bioceramic sealers are known to form hydroxyapatite upon contact with tissue fluids, creating a chemical bond between the sealer and the dentinal walls, thus enhancing the sealing properties and promoting periapical healing [4]. Several studies have reported that bioceramic sealers exhibit antimicrobial properties, promote mineralization and improve healing of periapical tissues compared to conventional sealers [5, 6]. Additionally, their ability to expand slightly upon setting further improves the sealing capability of root canal fillings [7]. Despite these advantages, the clinical effectiveness of bioceramic sealers in improving root canal healing requires further investigation through randomized controlled trials. Therefore, it is of interest to assess the effect of bioceramic sealers in enhancing root canal healing in comparison to conventional sealers.

## Materials and Methods:

## Study design and population:

This randomized controlled trial was conducted on 100 patients (aged 18-65 years) diagnosed with infected root canals at the Department of Endodontics. Inclusion criteria included patients with non-vital teeth requiring root canal treatment and the presence of periapical radiolucency. Patients with systemic conditions affecting healing (*e.g.*, diabetes, immunocompromised states), pregnant women and those with previously treated root canals were excluded.

## Sample size and randomization:

The sample size was determined based on previous studies showing a 15% difference in healing rates between bioceramic and conventional sealers, with a 95% confidence level and 80% power. A total of 100 participants were randomly assigned to one of two groups (n=50 per group) using a computer-generated randomization table:

[1] Group A: Treated with bioceramic sealer (EndoSequence BC Sealer, Brasseler USA)

[2] Group B: Treated with conventional sealer (AH Plus, Dentsply Sirona)

## Root canal treatment procedure:

All patients underwent standardized root canal therapy. After administering local anesthesia (2% lidocaine with epinephrine 1:100,000), an access cavity was prepared using sterile diamond burs. The root canals were cleaned and shaped using Pro-Taper rotary files (Dentsply Sirona) and irrigated with 5.25% sodium hypochlorite. Final irrigation was done with 17% EDTA followed by saline. The canals were dried with paper points. In Group A, bioceramic sealer was applied according to the manufacturer's instructions and the canal was obturated with gutta-percha using the single-cone technique. In Group B, conventional epoxy resin-based sealer was used with lateral condensation technique for obturation. Post-obturation radiographs were taken to confirm the quality of the root canal filling.

## Follow-up and outcome measures:

Patients were followed up at 3 and 6 months post-treatment. Clinical evaluations included assessment of pain using a visual analog scale (VAS), with scores ranging from 0 (no pain) to 10 (worst possible pain). Radiographic analysis was performed to evaluate periapical healing using periapical index (PAI) scores. The PAI is a 5-point scale where 1 indicates normal periapical structures and 5 indicates severe periapical radiolucency.

## Statistical analysis:

Data were analyzed using SPSS software (version X.X). Descriptive statistics were calculated for baseline characteristics. The chi-square test was used to compare healing outcomes between groups. Pain scores were analyzed using the Mann-Whitney U test. A p-value of <0.05 was considered statistically significant.

## Results:

A total of 100 patients (50 in each group) completed the study, with no dropouts. Baseline characteristics, including age, gender distribution and initial periapical index (PAI) scores, were comparable between the two groups (p > 0.05). At 3 months, the mean pain score in Group A (bioceramic sealer) was 1.8 ±0.5, while in Group B (conventional sealer) it was 2.5 ±0.7. By 6 months, Group A showed a mean pain score of 0.8 ±0.2 compared to 1.5 ±0.4 in Group B. The difference between the groups was statistically significant at both time points (p < 0.05) (Table 1). Radiographic analysis revealed significant differences in periapical healing between the two groups. At the 6-month follow-up, 90% of patients in Group A exhibited complete resolution of periapical lesions (PAI score of 1 or 2), compared to 75% of patients in Group B. The difference was statistically significant (p = 0.03) (Table 2). The radiographic analysis confirmed that patients treated with bioceramic sealer had fewer voids in the obturated canals compared to those treated with conventional sealers. The presence of voids was observed in 5% of cases in Group A and 15% of cases in Group B, indicating superior filling quality with bioceramic sealers (p = 0.02). In summary, bioceramic sealers demonstrated superior outcomes in both pain reduction and periapical healing compared to conventional sealers. These findings suggest that bioceramic sealers offer significant advantages in enhancing root canal therapy success.

## Discussion:

The findings of this study suggest that bioceramic sealers significantly enhance root canal healing compared to conventional sealers. The superior healing outcomes observed in patients treated with bioceramic sealers can be attributed to their unique properties, including biocompatibility, bioactivity and superior sealing ability. Bioceramic sealers have been widely reported to promote periapical healing by inducing the formation of hydroxyapatite and establishing a chemical bond with dentinal walls [1, 2]. This bioactive characteristic helps in creating a tight seal that prevents microbial leakage, thereby facilitating healing [3]. One of the most critical advantages of bioceramic sealers is their ability to expand slightly upon setting, enhancing their sealing ability. Studies have shown that this slight expansion improves the long-term success rate of root canal treatments by minimizing the occurrence of voids within the root canal [4, 5]. Additionally, bioceramic sealers' ability to form hydroxyapatite on the surface of dentin and sealant interfaces further enhances their effectiveness in promoting healing [6]. The findings in this study align with other research that demonstrates bioceramic sealers' ability to reduce periapical lesions more efficiently than conventional materials [7, 8]. In contrast, conventional sealers, such as epoxy resin-based sealers, offer strong physical sealing properties but lack the bioactive qualities needed to promote tissue healing and regeneration. While these conventional sealers can create an adequate seal, they may be prone to shrinkage over time, which can result in microleakage and reinfection of the root canal system [9, 10]. Previous studies have indicated that conventional sealers, such as AH Plus, may not offer the same long-term clinical success as bioceramic sealers due to these limitations [11, 12].

Pain reduction was another significant outcome in this study, with patients in the bioceramic sealer group reporting less postoperative pain compared to the conventional sealer group. Bioceramic sealers demonstrate superior clinical performance over traditional sealers in root canal therapy, with better pain reduction, faster healing, and higher success rates [13]. The ability of bioceramic materials to minimize inflammation and promote faster healing has been highlighted in various clinical studies [14]. Extrusion of bioceramic sealer iRoot SP does not adversely affect root canal treatment outcomes, demonstrating a high success rate and clinical safety [15]. The antimicrobial properties of bioceramic sealers also contribute to their superior clinical performance. Research has demonstrated that bioceramic sealers have intrinsic antimicrobial activity, which is effective against common endodontic pathogens, including *Enterococcus faecalis* [16, 17]. This antimicrobial action, combined with their superior sealing ability, prevents bacterial regrowth and reinfection, contributing to better treatment outcomes [18]. Furthermore, bioceramic sealers are less prone to shrinkage or dissolution over time, which may explain their superior radiographic performance in long-term studies [19]. In summary, bioceramic sealers outperform conventional sealers in several critical areas, including biocompatibility, sealing ability, pain reduction, antimicrobial properties and periapical healing. The results of this study further support the growing body of evidence recommending the use of bioceramic sealers for root canal therapy to improve clinical outcomes and long-term success rates [20]. Bioceramics, particularly MTA and bioceramic-based sealers, enhance endodontic outcomes through superior biocompatibility, sealing ability, and regenerative potential [21]. Bioceramic-based root canal sealers demonstrate bioactivity through mineralization and gene expression, with standardized *in vitro* and in vivo methods confirming their regenerative potential [22].

## Conclusion:

Bioceramic sealers provide enhanced root canal healing results than traditional sealers because they generate better pain reduction together with better periapical lesion resolution and better sealing properties and stronger antimicrobial behavior. These materials promote better tissue regeneration as well as long-term success because of their biological compatibility and active properties.

## Figures and Tables

**Table 1:** Comparison of pain scores at 3 and 6 months

**Time Point**	**Group A (Bioceramic Sealer)**	**Group B (Conventional Sealer)**	**p-value**
3 months	1.8 ± 0.5	2.5 ± 0.7	< 0.05
6 months	0.8 ± 0.2	1.5 ± 0.4	< 0.05

**Table 2:** Comparison of periapical healing at 6 months

**Healing Outcome**	**Group A (Bioceramic Sealer)**	**Group B (Conventional Sealer)**	**p-value**
Complete healing (PAI 1 or 2)	90% (45/50)	75% (38/50)	0.03
Partial healing (PAI 3 or 4)	10% (5/50)	25% (12/50)	0.03
